# Effects of Bushen Huoxue method for female with decreased ovarian reserve: A protocol for systematic review and meta-analysis

**DOI:** 10.1097/MD.0000000000022957

**Published:** 2020-10-23

**Authors:** Zhuoting Xie, Yin Li, Zehua Chen, Qiuyu Cao, Chunren Zhang, Yang Fei, Kunyin Li, Yongge Guan

**Affiliations:** a The Third Clinical Medical School; b The First Clinical Medical School, Guangzhou University of Chinese Medicine; c The Third Affiliated Hospital of Guangzhou University of Chinese Medicine; d The Fifth Clinical Medical School, Guangzhou University of Chinese Medicine, Guangzhou, Guangdong Province, China.

**Keywords:** Chinese medicine, decreased ovarian reserve, premature ovarian failure, protocol, systematic review

## Abstract

**Background:**

Decreased ovarian reserve (DOR) is a common reproductive barrier in female. Bushen Huoxue (BSHX) method of TCM is widely applied to treat DOR clinically. The purpose of this study is to provide a systemic and comprehensive evaluation of BSHX in the treatment of DOR.

**Methods:**

We have registered this protocol with OSF registry and the DOI is 10.17605/OSF.IO/QNUE2. We will search 4 English databases (PubMed, EMBASE, MEDLINE, Cochrane Library) and four Chinese databases (China national knowledge infrastructure database, Wanfang database, VIP and Superstar database) from their inception to August 10, 2020. Two authors will search and extract independently all related studies. RevMan 5.3 software will be applied to synthesize data.

**Results:**

The results of this study will be published in a scientific journal after peer-review.

**Conclusion:**

This systematic review will provide reliable evidences for clinicians, and help them make decisions in DOR management.

## 1 Introduction

Ovarian reserve function indicates the ability of follicles to growth in the ovarian cortex and to form fertilizable follicles.^[1]^ Along with ages, the recruited follicles are decreasing gradually in the ovary and the qualities of eggs are also declining. This process is called decreased ovarian reserve (DOR), which predicts the decline of fertility. DOR is an early stage of premature ovarian failure (POF).^[2]^ The main symptoms of DOR are menstrual changes including cycle disorder, shortened period, hypomenorrhea, even amenorrhea and infertility, accompany with perimenopausal period symptoms such as palpitation, insomnia, sweat, or waist ache.^[3]^ According to European Society of Human Reproduction and Embryology (ESHRE) guideline in 2016, the definition of DOR reaches:

(1) age <40, over one year normal menstrual cycle;
(2) DOR symptoms;
(3) abnormal ovarian reserve test.^[4]^

In clinic, DOR or POF is associated to recurrent miscarriage, unexplained infertility, repeated planting failure in IVF/ICSI, pre-eclampsia and many other fertility problems.^[5,6]^ Therefore, effective treatment of DOR is of great significance to infertile couples and improvements of female life quality.

There are many factors affecting the ovarian reserve, including aging, psychological disorders, autoimmune, metabolic diseases, infections, genetic abnormalities, cigarette smoking, chemotherapy, radiation and gynecologic surgeries.^[7]^ Hormone replacement therapy is the most common treatment for DOR patients, which can improve patients low estrogen status such as hot flashes, sweat or vaginal dryness, and help establish a normal hormonal cycle. Besides, for the infertile DOR patients, gonadotropins, recombinant LH, controlled ovulation induction, assisted reproductive treatment, growth hormone, coenzyme Q10, DHEA, and aspirin are effective therapies.^[8]^

Traditional Chinese medicine (TCM) has also been used to treat DOR for a long time. From TCM perspective, Shenqi dominates female reproductive abilities, and blood is the basis of female physiology. Shen deficiency and blood stasis are considered as the basic pathogenesis of DOR. Therefore, the Bushen Huoxue (BSHX) traditional medicine method is widely applied to treat DOR. Clinic trials have showed that BSHX can improve DOR symptoms effectively; up-regulate AMH and E2; down-regulate FSH; increase ovarian volume and antral follicle counts.^[9]^ Pharmacological researches have demonstrated that BSHX can balance female endocrine through down-regulating FSH level; prevent premature follicular apoptosis and adjust female immune system; improve pelvic blood microcirculation and increase blood supply for the ovary and uterus.^[10]^ Although BSHX has a good effect on DOR, there is lack of systematic review summarizing the efficiency of BSHX on DOR to give us evidences. Hence, according to evidence-based medicine principles, this study is to systematically review current clinical randomized controlled trials (RCTs) to assess the effectiveness of the BSHX in the management of DOR.

## 2 Methods

### 2.1 Registration

This protocol has been submitted and registered with the Open Science Framework (OSF, https://osf.io/). The registration DOI is 10.17605/OSF.IO/QNUE2. We will perform this work following the guidelines of Preferred Reporting Items for Systematic Reviews and Meta-Analyses (PRISMA-P).

### 2.2 Inclusion criteria

#### 2.2.1 Type of studies

In this review, all clinical randomized controlled trials (RCTs) that explore BSHX method in the treatment of decreased ovarian reserve (DOR) will be included. Experimental researches, reviews, and retrospective studies will not be considered.

#### 2.2.2 Types of patients

All patients in this review are diagnosed with DOR. There will be no limitation about races, region, and other factors.

#### 2.2.3 Types of interventions

Patients in the treatment group were treated with BSHX alone or in combination with conventional hormone therapies. Patients in the control group were intervened with no treatment, placebo, and conventional hormone therapies.

#### 2.2.4 Types of outcomes

##### 2.2.4.1 Primary outcomes

The primary outcome of this review will focus on female menstruation situations and perimenopausal period symptoms. Menstruation situations are measured by cycles, periods and menstrual volumes. Perimenopausal period symptoms are measured by the scale of hot flashes, sweating, bone aches. An outcome was reported if > 1scale. Bigger number indicates higher scale in the ranking order.^[11]^

##### 2.2.4.2 Secondary outcomes

The secondary outcome of this review will focus on basal levels of following hormones:

FSH
LH
E2
FSH/LH
AMH
AFC
OVARIAN VOLUME.

### 2.3 Search strategy

We will search following electronic databases, including 4 English databases (PubMed, EMBASE, MEDLINE, Cochrane Library) and 4 Chinese databases (China national knowledge infrastructure database, Wanfang database, VIP and Superstar database). The following MeSH search headings or keywords in Title/Abstract are used: “bushen huoxue” or “yishen huoxue” or “zishen huoxue” or “tonifying kidney AND activating blood” AND “ovarian dysfunction” or “decreased ovarian reserve” or “diminished ovarian reserve” or “premature ovarian insufficiency” or “premature ovarian failure” AND “randomized study” or “randomized controlled trial” or “controlled study”. This search will be conducted from inception to August 10, 2020. Two authors (ZX and YL) will search and extract independently all related studies.

### 2.4 Study selection and data analysis

#### 2.4.1 Study selection and data collection

**Figure 1:**
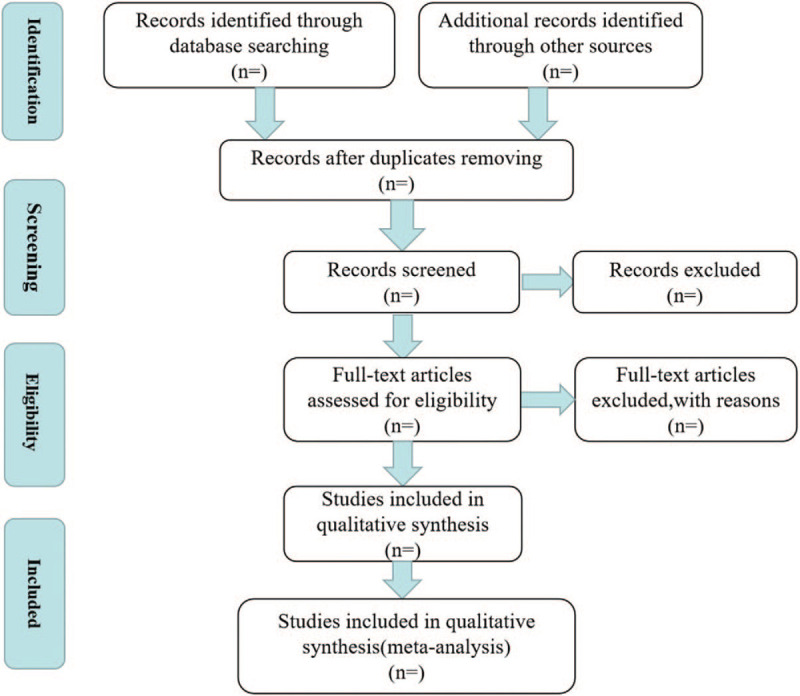
Flow diagram of study selection.

Searched articles will be included and collated by EndNote X9.0 (Stanford, Connecticut, https://endnote.com). Two authors (ZX and YL) will read the title and abstract independently to find related articles, then determine the eligible articles by reading through full articles. The following data from each study will be extracted: first author, year of publication, study design, sample sizes, inclusion and exclusion criteria, interventions, and the reported outcome. Repeated studies by the same authors will be kept for one. Different opinions will be discussed and resolved through consulting another research member. The final included articles will be filed in an EXCEL list. A PRISMA flow diagram has been drawn to summarize the study selection (Fig. 1).

#### 2.4.2 Assessment of risk of bias

According to the Cochrane Handbook for systematic reviews of interventions (V.5.1), the assessments of the studies are divided to “High risk,” “Low risk,” or “Unclear”. The risk of bias of studies will be assessed from 7 items: random sequence generation, allocation concealment, blinding of participants, blinding of outcome assessment, incomplete outcome data, selective reporting, and other issues. Two authors (ZX and YL) will conduct the evaluations about the methodological qualities of studies independently. The inconsistencies will be discussed and resolved through consulting another research member.

#### 2.4.3 Measures of treatment effect

For continuous variable data, the standardized mean difference (SMD) or mean difference (MD) with 95% confidence interval (CI) will be utilized to assess treatment effect. For dichotomous data, the risk ratio (RR) with 95% CI will be used.

#### 2.4.4 Assessment of heterogeneity

Chi-squared test and *P* value will be used in assessment of heterogeneity. When *I*^*2*^ > 50% and *P* < .05, it suggests that there is a significant statistical heterogeneity between two groups. When *I*^*2*^ ≤ 50% and *P* ≥ .05, it indicates that there is no significant statistical heterogeneity between 2 groups.

#### 2.4.5 Assessment of reporting biases

When included trials in this review are more than 10, the funnel plot and statistic test will be used to evaluate the reporting bias. The Egger test and Begg test are common statistic tests to assess reporting biases. *P* < .05 indicates publication bias while *P* ≥ .05 suggests no significant publication bias.

#### 2.4.6 Data synthesis

RevMan 5.3 software will be applied to synthesize data. According to the result of heterogeneity assessment, a fixed effect model is appropriate for the result which there is no statistical heterogeneity, and a random effect model is appropriate for the result which there is a significant statistical heterogeneity. Furthermore, subgroup analysis or sensitivity analysis will be used to explore the heterogeneity reasons.

#### 2.4.7 Subgroup analysis

When there is a significant statistical heterogeneity, we will use a subgroup analysis to explore the possible heterogeneity reasons. The following reasons will be taken into consideration: races, age, subtypes of the disease, BSHX therapeutic schedules.

#### 2.4.8 Sensitivity analysis

To evaluate the robustness and reliability of the results in a meta-analysis, we need to perform sensitivity analysis. The common methods of sensitivity analysis include altering inclusion criteria especially for those controversial studies, excluding low quality studies, applying different statistical models to analyze data. You can reestimate and compare the results of this meta-analysis after you have taken one sensitivity analysis method. If there is no significant change in the results, it indicates that the sensitivity is low and the results are robust and reliable.

#### 2.4.9 Grading of the evidence

The GRADE grading system is widely used in systematic evaluation. In this system, randomized controlled trials (RCTs) are defined as high-quality evidence and observational studies as low-quality evidence. The evidence grading will be promoted and demoted for some reasons such as reporting biases, indirectness, dose-response. Finally, the evidence grading will be determined to four different levels: high, medium, low, very low.

#### 2.4.10 Ethics and dissemination

It is not applicable for this systematic review and meta-analysis to require an ethical approval since this study is not involving with individual patient data. Besides, this review will be disseminated in peer-review journals.

## 3 Discussion

With the improvement of economic and educational level, female childbearing age is becoming delayed. DOR has become a general pressure.^[12]^ In China, more and more gynecologists and reproductive medicine specialists take traditional Chinese medicine as an effective complementary therapy for DOR patients, especially for those have needs of fertility. However, many of them usually have no idea to choose which Chinese medicine method to manage DOR patients. The BSHX is a classical method to treat DOR. Since there is no systematic review about BSHX method for DOR, we hope that this systematic review will provide reliable evidences for clinicians, and help them make decisions in DOR management. Besides, this study can also promote the progress of traditional medicine research.

## Author contributions

**Conceptualization:** Zhuoting Xie, Zehua Chen, Chunren Zhang.

**Data curation:** Zhuoting Xie, Yin Li, Qiuyu Cao.

**Formal analysis:** Zhuoting Xie, Yin Li, Qiuyu Cao.

**Funding acquisition:** Kunyin Li, Yongge Guan.

**Investigation:** Zhuoting Xie, Yin Li, Yang Fei.

**Methodology:** Zhuoting Xie, Yin Li, Yang Fei.

**Software:** Zhuoting Xie, Zehua Chen, Chunren Zhang.

**Supervision:** Kunyin Li.

**Writing – original draft:** Zhuoting Xie.

**Writing – review & editing:** Zhuoting Xie, Yongge Guan.
